# A systematic review of the theory of planned behaviour interventions for chronic diseases in low health-literacy settings

**DOI:** 10.7189/jogh.13.04079

**Published:** 2023-09-08

**Authors:** Biswajit Paul, Richard Kirubakaran, Rita Isaac, Marshall Dozier, Liz Grant, David Weller, RESPIRE Collaboration

**Affiliations:** 1 Christian Medical College Vellore, India; 2 University of Edinburgh, UK

## Abstract

**Background:**

Due to their chronicity, prolonged morbidity, and high mortality, chronic respiratory diseases (CRDs) pose a huge burden of disease globally, primarily among low- and middle-income countries. Most of these diseases can be controlled by early diagnosis and treatment, correct practice of medications, regular follow-up, and avoidance of risk factors, which involves a change in health behaviour among patients. The theory of planned behaviour (TPB) has been proven to be effective and has been used increasingly as a behavioural framework for designing and evaluating behaviour change interventions, although most such studies were on affluent populations and from the global north. We aimed to collate evidence of TPB-based behavioural interventions in low health literacy settings for its effectiveness and feasibility by conducting a systematic review (SR).

**Methods:**

We followed Preferred Reporting Items for Systematic Reviews and Meta-Analyses (PRISMA) 2020 guidelines in conducting and reporting this study. We selected interventional studies using at least two constructs of TPB for behaviour change in chronic disease patients and conducted in LMICs, used the PICO framework, and exported the retrieved studies through the Endnote software. We evaluated the studies using the Risk of Bias (RoB) 2 and Risk of Bias in Non-randomised Studies – of Interventions (ROBINS-I) tools.

**Results:**

We retrieved and reviewed the titles and abstracts 4281 titles and abstracts, identifying 186 articles for further detailed screening. Eleven studies met the criteria for a standardised independent full-text screening by two authors and four were selected for narrative synthesis. All studies were from urban settings, with established feasibility and fidelity; all interventions were effective in changing health behaviour and TPB constructs and provided structured education to participants in the intervention group (either face-to-face and through group education). Three studies had some concerns/moderate risk of bias and one had high risk of bias.

**Conclusions:**

All studies demonstrated effectiveness, feasibility, and fidelity of TPB interventions in LMIC settings, although most were of moderate quality. Further studies should gather definitive evidence and prove their feasibility and utility in LMICs.

**Registration:**

PROSPERO CRD42018104890.

Chronic diseases are conditions which persist over a long period of time, have long-lasting effects, or even may develop over time. They consequently lead to high rates of morbidity and mortality among the affected individuals. Various organisations such as the United States National Centre for Health Statistics (USNCHS) and Centres for Disease Control and Prevention (CDC) define chronic diseases differently in terms of duration, ranging from more than three months to more than a year [1]. They also differ regarding conditions categorised as chronic diseases [2-5], but they nevertheless affect all age groups and all regions worldwide. Chronic diseases, including non-communicable diseases, are associated mainly with older age groups and cause most of the premature deaths ( ~ 85%) in low- and middle-income countries (LMICs) [3]. Health behaviour can modify or influence many of the risk factors, leading to better management of chronic diseases and improving compliance to treatment [6].

Health behaviour encompasses actions taken by individuals which affect their physical and mental health and quality of life [7]. Human behaviour influences health, potentially underlying more than 50% of illness [8,9]. They also play a role in communicable diseases such as COVID-19, where human behaviours (e.g. use of face mask, social distancing, handwashing, and vaccination) influence disease prevention and pandemic response [10]. Health behaviours play a major role in health promotion, disease prevention, adherence to medications, disease control, and quality of health care delivery, but their role in the prevention and control of chronic diseases is especially prominent [11-13].

Behaviour change interventions can target individual, organisational, community, and population levels (with any intervention delivered at one level possibly impacting others), with those targeting several levels simultaneously and consistently being the most effective [14,15]. A change in health behaviour warrants an understanding of the existing health behaviour and transformation of this knowledge into effective strategies for behaviour improvement through developing or using theories. An understanding of theories of behaviour change is important to designing interventions that yield desirable changes and the capability to use them skillfully in research and practice [16]. The UK Medical Research Council (MRC) emphasises the use of a theory in intervention design; theory improves intervention effectiveness and allows for replication [17].

The theory of planned behaviour (TPB) [18,19] states that behaviour is an outcome of individual beliefs related to that behaviour which determines the attitude towards the behaviour, the subjective norms prevalent for that behaviour, and the perceived behavioural control leading to an intention to perform that behaviour (TPB constructs), with intention being the most important determinant and immediate antecedent to a particular health behaviour. TPB was subsequently generated from the theory of reasoned action (TRA) when behavioural control was added as an important construct influencing health behaviour along with attitude towards the behaviour and subjective norms. TPB assumes a causal chain that links beliefs (behavioural, normative, and control beliefs) to behavioural intentions and behaviours via attitudes, subjective norms, and perceived behavioural control, and provides a systematic method to identify the most important issues for a person’s decisions to perform a specific behaviour. Because many important beliefs and attitudes are changeable, they are ideal targets for subsequent interventions. TPB has been increasingly used as a behavioural framework for designing and evaluating behaviour change interventions and their outcomes [20-22].

There is a lack of data on changes in health behaviour resulting from TPB-based interventions in chronic diseases and their applicability in different settings, particularly LMICs. Three other systematic reviews (SRs) on TPB-based interventions have been conducted earlier. SRs by Hardeman et al. [23] conducted in 2001 examined TPB interventions to change health behaviour on any population where TPB has been applied without any mention of chronic diseases. It mainly measured process and outcome variables and to predict intention and behaviour. In 2015, Rich et al. [24] examined the role of TPB in predicting adherence in people with a chronic condition. Their research suggested that TPB made a useful contribution to our understanding of adherence in chronic illness; it measured the types of adherence behaviours and the effects of the TPB constructs on adherence behaviour. However, it did not examine the settings in which the interventions were delivered or had any reference to health literacy and excluded studies with populations considered to be at risk of chronic disease (e.g. sedentary adults). Steinmetz et al. [25] in 2016, incorporated a three-level meta-analysis to establish that interventions based on TPB were effective in changing behaviour. The mean effect size was 0.50 for antecedent variables (behavioural, normative, and control beliefs, attitude, subjective norm, perceived behavioural control and intention); types of conditions were not specified.

Most studies on TPB were done in the western world among high-income populations, with predictive studies focusing on affluent, young, and fit individuals [22,26,27]. Although some researchers have designated the TPB as “Western” and fit for well-educated population [28], others have encouraged its cross-cultural application and a further understanding of the behaviour from the study population’s perspective [29,30]. It is important to study beliefs underlying TPB constructs specific to the behaviour and population being investigated. We conducted a SR on chronic disease patients in resource-poor, low health literacy settings using TPB-based behavioural interventions to determine the effectiveness of such interventions in these population settings, test their feasibility, and informd their development and implementation in LMICs. Our objectives were:

- To determine the feasibility and fidelity of TPB based interventions in low health literacy settings of LMICs
- To determine the impact of TPB based interventions for behaviour change among chronic disease patients on health outcomes (improvement of symptoms, quality of life), individual behaviour outcomes (preventive behaviours, lifestyle changes, adherence to therapy/medications, regularity of follow-up/treatment, care seeking), TPB constructs like attitude towards behaviour, subjective norms and perceived behavioural control
- To describe the TPB interventions in terms of their development methods, types of intervention used, time frame/modes of delivery and the settings of such delivery (urban, rural, male, female, socio-economic status (SES)).

## METHODS

### Protocol and registration

We followed the Preferred Reporting Items for Systematic Reviews and Meta-Analyses (PRISMA) 2020 guidelines [31] in conducting this study, developing a protocol and registering it with the University of York Centre for Reviews and Dissemination and the International Prospective Register of Systematic Reviews (PROSPERO) database (CRD42018104890). The protocol was also published elsewhere [32].

### Types of studies

We included randomised controlled trials, clustered randomised community trials, and quasi-experimental studies/non-randomised trials [33], as well as studies comparing the intervention with a comparison group (including placebo, treatment as usual/standard care, or comparison with a different intervention than in the designated intervention group) and studies with more than one intervention group or within subjects across time (i.e. controlled before and after studies).

We excluded case-control studies, cohort studies, cross-sectional studies, reviews, case reports, case series, and animal studies, as well as studies on health behaviour change which do not mention TP or at least two constructs of TPB among four (attitude towards health behaviour, subjective norms, perceived behavioural control and intention) or other psychological theories. We also excluded studies undertaken on healthy individuals with a purely health promotion focus.

We did not set any restrictions on language in order to include various TPB based interventions in different settings, while still focusing specifically on LMICs, but also because our initial search retrieved few studies in English.

### Types of participants

Participants were adults aged ≥18 years with one or more chronic diseases [3], including cardiovascular diseases, cancers, chronic respiratory diseases, diabetes, hypertension, obesity, chronic mental illness like Alzheimer disease, chronic bone or joint disease like osteoarthritis, and human immunodeficiency virus (HIV)/acquired immunodeficiency syndrome (AIDS). We excluded studies on healthy populations and pregnant women.

### Types of interventions and control/comparator

We considered health or educational interventions using the constructs of TPB (attitude towards the behaviour, subjective norms, perceived behavioural control and intention) to change health behaviour (lifestyle/preventive behaviours/adherence to medications/health seeking behaviour) for inclusion, as well as interventions using underlying beliefs to the TPB constructs (behavioural, normative, and control beliefs) and terms like evaluation of outcomes, motivation to comply, and perceived power, as informed by a scoping review of literature. As mentioned earlier, we included TPB-based interventions based if they used at least two of the constructs and/or underlying beliefs, and if they were conducted either on individuals or on groups (hospital- or community-based).

We further included interventions applied on patients with chronic diseases (defined above) and on LMICs with low health literacy populations. Health literacy has been defined as “the degree to which individuals can obtain, process, and understand the basic health information and services they need to make appropriate health decisions” [34] and influence health behaviours and outcomes [35-37]. Health literacy in LMICs is lower than that measured in the USA and other high-income countries (HICs) due to lower general income and education of their populations [38-40]. We categorised LMICs per the World Bank 2017 definition (per capita income) [41], using it as a proxy for populations with low literacy, health care, and income/socio-economic status, i.e. low health literacy and health resources.

The control or the comparator was health or educational intervention not based on any psychological theory, health education based on psychological theory other than TPB, or treatment as usual without any education. For included controlled before-and-after studies, the study group which was used as control with usual health education or no education was considered as the intervention group following TPB-based intervention, and these outcomes were compared.

### Outcomes

Our primary outcome was change in health behaviour (including preventive behaviours, lifestyle changes, adherence to treatment, and care seeking) following an intervention. The World Health Organization (WHO) defined adherence as as “the extent to which a person’s behaviour – taking medication, following a diet, and/or executing lifestyle changes, corresponds with agreed recommendations from a health care provider” [42]. Other key study elements were study setting, constructs of TPB which influenced health behaviour change, time-frame of such interventions, its feasibility and fidelity (i.e. recruitment rates) and integrity, completion and follow-up rates, and adherence to time frame. Other important elements were the mode of delivery of the intervention, type of health providers implementing the intervention, and patient satisfaction. These characteristics informed researchers for building a feasible, effective, and appropriate intervention for a LMIC setting.

### Information sources and search strategy

We searched MEDLINE, Embase, Cochrane Library, PsychINFO, Web of Science, Scopus, CINAHL, ProQuest databases (ProQuest Sociology and ProQuest Social Sciences), Global Index Medicus, Bibliography of Asian Studies, and IndMED for relevant studies published between 1980 and 2019. We used keywords, truncation, and medical subject heading (MeSH) terms, combining them with database-specific syntax, parentheses, Boolean operators, and field codes [43]. We stratified the search into five categories: theory of planned behaviour, trials, health behaviour or adherence, chronic disease, and LMICs, based on previous research, theory, and practice. The first category used TPB/TRA and related terms to search for studies based on it, as it was the central to the review objective. The second category used terms related to randomised and non-randomised trials, longitudinal studies, and feasibility studies. The third category comprised health behaviour or adherence (included in our primary objective) and used keywords like behaviour change, health seeking behaviour, adherence or compliance. The fourth category specified on chronicity of the disease or condition and used chronic disease and chronic respiratory diseases. The fifth and final category searched for studies from LMICs and used search terms like low income, underserved population, less developed, developing countries, and specific country names or regions (Appendix S1 in the **Online Supplementary Document**). We also conducted an additional web-based search using titles and phrases.

### Study selection and data collection process

We exported all relevant studies using EndNote, versions X9 and 20 (Clarivate, London, IL), which we also applied for screening, deduplication, and general management. One author (BP) conducted the searches, exported the results, and deduplicated the retrieved studies. Two reviewers (BP and RK) screened their titles and abstracts following pre-defined inclusion criteria to assure correctness and avoid omission of relevant records (**Table 1**). Discrepancies were resolved by discussion with MD or DW. The full-text screening followed a similar process, with BP and RK reviewing the studies using pre-defined inclusion/exclusion criteria (**Table 1**). All studies were reviewed and confirmed by MD, RI, DW, or LG prior to inclusion in the final synthesis. Studies were included if their full text was available by the cut-off date of March 2019.

**Table 1:** Inclusion and exclusion criteria for screening

Serial number	Characteristic	Criteria for inclusion/exclusion
1	Population	The selected population should be adults above 18 y of age, either males or females or mixed population and should not be Caucasian. If any population <18 y of age is defined as adult as per the country’s classification, then it will be accepted.
2	Disease /Condition	Any chronic disease or condition is acceptable for inclusion since the search criteria involves population with any chronic disease while excluding research studies involving healthy population or pregnant women. Thus, globally recognised terminologies of disease classification are used, i.e. non-communicable diseases like cardiovascular disease, stroke, diabetes, hypertension, obesity, chronic bone or joint disease like osteoarthritis, chronic mental illness and cancer are included along with people suffering from HIV/AIDS
3	Intervention	Educational or health intervention for behaviour change using TPB will be included; studies using multiple theories for behaviour change will be included provided there are measurable outcomes effected by TPB. Studies considering at least two constructs of TPB will be considered. The constructs of TPB are attitude towards health behaviour, subjective norms and perceived behavioural control and intention; of course, underlying beliefs will also be considered for inclusion.
4	Control/comparator	Comparison will include health or educational intervention for behaviour change not based on any psychological theory or health education using behavioural theories other than TPB. Individuals or groups getting treatment as usual without any structured health education are also eligible as controls.
5	Study design	Only interventional studies measuring effect after a period of intervention will be included; this includes clinical trials, randomised and non-randomised trails, cluster randomised and community trails, before and after studies and longitudinal and feasibility studies with TPB intervention.
6	Setting	Population belonging to LMICs as defined by World Bank 2017 per capita income is included for the selection. The reason behind this selection is to use it as a proxy for population with low literacy, health care and income/socio-economic status, i.e. low health literacy and health resource.
7	Outcomes	Any measurable change in knowledge, attitude and health behaviour specific to a chronic disease condition will be evaluated which will include measurable difference in disease awareness, frequency and/or duration of exercises, drug adherence and self-care.

HIV – human immunodeficiency virus, AIDS – acquired immunodeficiency syndrome, TPB – Theory of planned behaviour, LMIC – low- and middle-income countries

### Data items, summary measures, synthesis and analysis of results

We adapted data extraction form from the Cochrane Collaboration data collection form of randomised controlled trials (RCTs) and non-randomised studies (NRS) [44]. BP extracted the data, MD re-checked it and DW and LG reviewed it for confirmation. We extracted descriptive information from each intervention, including the study name, author(s), place and year of study, study design features (e.g. data collection points, inclusion of a control group or not), and socio-demographic characteristics (including age, gender, and education/literacy levels) (**Table 2**). To assess the effect of the interventions, we extracted the name of the outcome measure(s), reported value(s) for intervention effectiveness (i.e. *P*-value, effect size), and prior research that provided a narrative commentary on study design methods that may influence the generalisability of study effects. We considered outcomes to be statistically significant if a *P* < 0.05 [49] was reported for the quantitative analyses. For combining and reporting the results of narrative synthesis, we inspected each study’s methods and outcomes and categorised them by the following key themes: study setting, feasibility and fidelity of the intervention, effectiveness of the study, methods of health education/intervention delivery, health providers for study implementation, and outcomes (change in knowledge, attitude, behaviour or adherence, i.e. increased knowledge, improved attitudes or change in health behaviour or quality of life) (**Table 3**). We did not conduct a meta-analysis due to substantial heterogeneity for construct measurement and operationalisation (e.g. improvement of healthy lifestyle vs improvement in quality of life). We also did not conduct additional subgroup or sensitivity analyses due to the small number studies included.

**Table 2:** Descriptive information of the four included studies

Study	Place of study; year of study	Study design; duration	Sample characteristics
Saffari et al. [45]	Tehran, Iran, 2016	Randomised controlled trial with intervention (I) and control groups (C), I – TPB based intervention, C – routine treatment, no educational intervention; May - September 2016, total of five months	n = 120 (I = 60; C = 60), mean age = 55.8 ± 8.9 y, gender: females = 91 (75.8%), education: illiterate/elementary = 68 (56.7%), high school/sary = 27 (22.5%), university = 25 (20.8%), economic status: good = 12 (10%), not good, not bad = 90 (75%), bad 18 (15%), married = 112 (93.3%), employed = 10 (8.3%)
Askari et al. [46]	Fasa City, Iran; 2016	Randomised clinical trial with intervention and control groups, I – educational intervention including two constructs of TPB and some other components; training sessions (two/week), a total of eight sessions; physical activity (walking for three times a week of 20-min duration each), C – not clear; coordination performed with medical staff at centre; duration – not specified; one month of intervention and endline evaluation after three months	n = 108 (I = 54; C = 54), mean age: I = 66.45 ± 3.40 y, C = 67.11 ± 3.25 y, gender: females (I = 36 (66.66%); C = 34 (62.97%)), education: illiterate/primary school (I = 14 (25.8%); C = 17 (31.5%)), high school/sary (I = 34 (63%); C = 30 (55.5%)), college (I = 6 (11.3%); C = 7 (13%))
Jeihooni et al. [47]	Fasa city, Iran; 2016-17	Quasi-experimental trial with intervention and control groups, I – educational intervention based on TPB, C – Routine health education by nurses; duration – June 2016 to May 2017, one year	n = 100 (I = 50; C = 50), mean age: I = 52.80 ± 6.71 y, C = 51.65 ± 6.90 y, gender: females (I = 19 (38%); C = 20 (40%)), education: illiterate/elementary (I = 5 (10%); C = 5 (10%)), Junior High school/high school (I = 33 (66%; C = 35 (70%)), College (I = 12 (24%); C = 10 (20%)), married: I = 45 (90%), C = 43 (86%), employed: (I = 29 (58%); C = 28 (56%))
Karimy et al. [48]	Bandar Abbas, Iran; 2014	Controlled clinical trial with intervention and control groups, I – TPB based educational intervention, C – No educational intervention, routine nursing advise on discharge; duration – not defined; Intervention period of one month and the evaluation at one month and three months after the completion of intervention	n = 80 (I = 40, C = 40), mean age: 51.5 + 7.07 y, gender: females n = 27 (33.75%), education: illiterate/elementary (I = 18 (45%); C = 17 (42.5%)), Middle school/high school (I = 13 (32.5%; C = 14 (35%)), University (I = 9 (22.5%); C = 9 (22.5%), married: I = 35 (87.5%); C = 34 (85%), employed: (I = 10 (25%); C = 12 (30%))

I – intervention, C – control group, TPB – Theory of planned behaviour

**Table 3:** Summary of findings – feasibility and outcome measures

Study	Study setting	Feasibility and fidelity	Effectiveness/Outcomes achieved	Intervention description – methods used, mode of delivery and health providers involved
Saffari et al. [45]	1. Hospital based study 2. Urban setting (city)	Feasibility – yes, 1. Recruitment rates – 82.2%., 2. Integrity of intervention delivery – Present Structured intervention with specific activities with timeline; follow up done for three months after intervention, 3. Completion rates 89.2%, 4. Completion within stipulated time – yes, fidelity – yes (very good), Intervention was completed, Overall dropout rate – 10.8% (completion rate in intervention arm = 88.3%; in control arm = 90%)	Objectives achieved, 1. Significant improvement in quality of life measured through three different scales within intervention group and between intervention and control groups, 2. Significant improvement in some clinical outcomes within intervention group and between intervention and control groups, 3. Significant improvement in all five TPB constructs within intervention group and between intervention and control groups	Educational intervention was given over seven group sessions (groups of eight to 10), each about 60-90 min; lectures and interactions, brainstorming, group discussion, role play, listing by participants about their control beliefs, educational film, CD-ROM and booklet about preventive lifestyles and adherence to treatment; face to face with direct interaction of the trainer with the participants in multiple sessions; health provider – trainer – no specifics given
Askari et al. [46]	1. Community based study (participants chosen from list at diabetic centre and available at community during selection; place of training/educational sessions – not mentioned; follow-up at homes by telephone), 2. Urban setting (city)	Feasibility – yes (excellent), 1. Recruitment rates – 100%, 2. Integrity of intervention delivery – Present; Intervention was provided through eight sessions over a one month period and follow-up was done at four weeks and eight weeks after intervention through telephonic calls. Training was for 70 min per session and given through lecture, questions and answers and group discussion, 3. Completion rates – 100%, 4. Completion within stipulated time – yes, fidelity – yes (excellent) All participants selected in the study completed the study with no dropouts	Objectives achieved, 1. Significant improvement in TPB constructs – (attitude and subjective norms) and behaviour (nutrition and jogging) within intervention arm and between two groups, 2. Significant improvement in biochemical indices (laboratory outcomes) within the intervention arm and between groups	Training in eight sessions (two/week), each of 70 min. duration: lectures, question and answer sessions and group discussions. Some images or visual content was also provided. Pamphlets were given to families and relatives, hybrid – face to face training sessions with telephonic follow-up, researchers
Jeihooni et al. [47]	1. Community based (patients were selected from the hospital list of admitted myocardial infarction patients and chosen from the community during selection), 2. Urban setting (city)	Feasibility – yes, 1. Recruitment rates – not mentioned, 2. Integrity of intervention delivery – present, structured educations sessions (one per week for eight weeks covering TPB constructs and nutrition: each of the five groups received same education for eight sessions, 3. Completion rates 100% (all completed), 4. Completion within stipulated time – yes, time frame was adhered to, fidelity – yes (excellent). All participants were available for endline evaluation and went through the intervention, no dropouts.	Objectives achieved, 1. Significant improvement in all five TPB constructs – (attitude, subjective norms, perceived behavioural control, intention and behaviour/lifestyle within intervention arm and between two groups after intervention period, 2. The TPB constructs (attitude, subjective norms and perceived behavioural control) contributed to 39.6% of intention to change lifestyle.	Eight sessions (one/week) of 55-60 min duration for participants divided into five groups of 10 each, group discussion, educational movies, role playing, answering questions of participants on common beliefs; one lecture session by a cardiologist, group discussion and step by step teaching to adopt a healthy lifestyle, face to face with direct interaction of the educator and nutritionist with the participants in multiple sessions, educational intervention – educator, nutrition advise by – nutritionist, one session by an cardiologist (on subjective norms)
Karimy et al. [48]	1. Community based study (patients were selected from the hospital list of admitted MI patients and chosen from the community during selection), 2. Urban setting (city)	Feasibility – yes, 1. Recruitment rates – not mentioned (participants were replaced if refused to consent to achieve sample size), 2. Integrity of intervention delivery – present; structured sessions for all participants in three groups with same content; methods used were same; instructor for these sessions not mentioned (same or different), 3. Completion rates – 86.25%, 4. Completion within stipulated time – yes, completed within time frame, fidelity – yes (very good). The overall dropout rate was 13.75%. The completion rate for intervention arm was 90% and for control arm was 82.5%.	All objectives were achieved, 1. Significant improvement in healthy lifestyle in intervention group following intervention., 2. Improvement in TPB constructs of attitude, subjective norms, perceived behavioural control and intention in the test group as compared to control group.	Four 50-min sessions (one/week) in three groups of 10-15 participants, group discussions, film screenings, questions and answer sessions, face to face with direct interaction of the instructor and nutritionist with the participants in multiple sessions, educational intervention – instructor, nutrition advise by – nutritionist

TPB – Theory of planned behaviour

### Risk of bias within and across studies

We evaluated the risk of bias of each study using either the Risk of Bias 2 (RoB 2) tool for randomised trials [33] or Risk of Bias in Non-randomised Studies – of Interventions (ROBINS-I) tool [50] for non-randomised studies. We assessed the studies by the criteria suggested in the Cochrane Handbook of Systematic Reviews of Interventions [44], categorising them as having “low”, “some concerns”, or “high” risk of bias. This bias can arise from the randomisation process, deviations from intended interventions, missing outcome data, the outcome measurement, and bias in selection of the reported result. We also provided an overall judgement based on the assessment for each domain.

## RESULTS

### Descriptive results

We reviewed 4281 titles and abstracts (3697 from ProQuest Sociology, 257 from Scopus, 237 from Cochrane, 41 from EMBASE, 24 from MEDLINE, nine from Web of Science, seven from Global Health, four from CINAHL, and two from Psych INFO). We identified three articles via the web search, one during scoping review and two following the database search. Ninety-five articles were removed through deduplication. We excluded 4003 during the title and abstract screening. We then identified 186 articles for a further detailed screening; 11 met the criteria for a standardised independent full-text screening by two authors.

**Figure 1:**
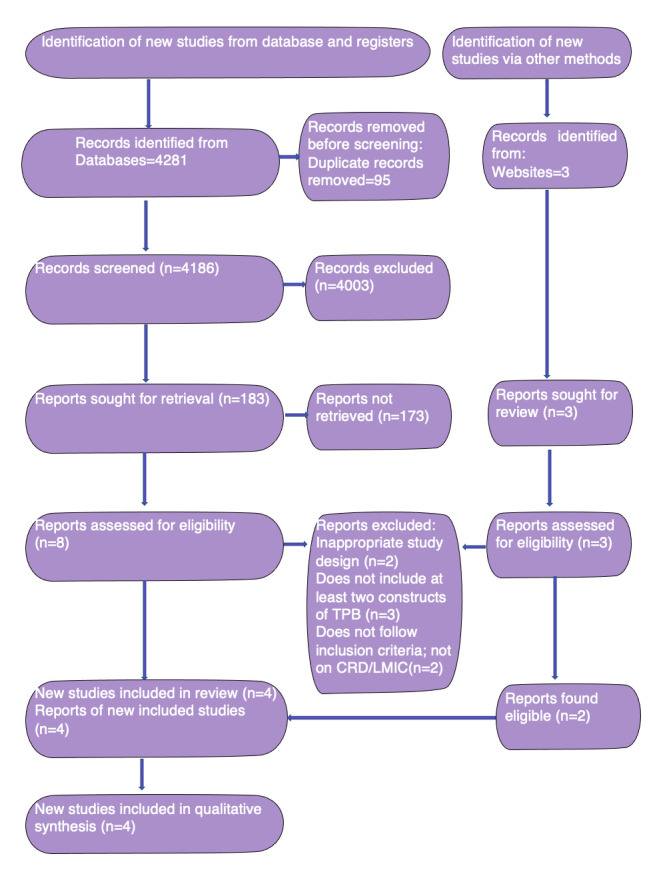
The PRISMA 2020 flow diagram.

A further seven articles were excluded because they did not meet the inclusion criteria on at least one level. Three articles [51-53] did not use TPB for changing behaviour; one [51] used coping and action planning, one [52] used Gollwitzer’s Implementation Intention Theory to change intention to behaviour for medication adherence, and the third [53] used a TPB questionnaire for evaluation of eating behaviour but did not use TPB for health intervention. A further two studies [54,55] used TPB, but were cross-sectional in design and did not fulfil the inclusion criteria for interventions. The sixth study [56] included healthy individuals and the intervention was for health promotion, rather than patients with chronic disease. The last study [57] was not conducted in a LMIC. Of the three articles identified by manual web search, one was a conference abstract of an ongoing study and was not included in the final synthesis, while the remaining two were already included in the eleven studies that underwent full-text screening. Finally, four articles were included for narrative synthesis following a full agreement and subsequent review of their full texts (**Figure 1**) [58].

### Study characteristics

The four studies [45-48] included 408 participants, of whom 227 were female and 181 male. Only one study reported economic status [45] (**Table 2**). All four studies reported the participants’ education status; three [45,47,48] also reported their marital and employment status. The interventions were delivered across a range of chronic disease, including cardiovascular disease patients with previous myocardial infarction (n = 2), diabetes (n = 1), and knee and hip osteoarthritis (n = 1). Three studies were randomised controlled trials [45,47,48] and one was a non-randomised (quasi-experimental) trial [46]. All studies were from Iran, categorised as an LMIC per World Bank definition [41]. A TPB-based educational intervention was given to the intervention group in all four studies. Routine education was provided for control groups in three studies [45,47,48], while the status was unclear for one study [46].

### Results from systematic review

Studies selected for inclusion were published between 2014 and 2017. Except for one [45], all were community-based (**Table 3**). All the studies demonstrated feasibility and fidelity in terms of recruitment, integrity of intervention delivery, and high completion rates (>80%); all such studies could be completed within the stipulated time designated for the trial. The effectiveness of such trials was evaluated in terms of achievement of target objectives, e.g. improvement of quality of life, significant improvement in clinical or laboratory outcomes and improvement in TPB constructs, post-intervention. All four studies showed that they were effective in improving TPB constructs and other parameters as set forth in the study protocol. This systematic review also informed us that the educational intervention packages were mostly given as group education, each session lasted for 50-90 minutes; usually given one per week except for one [46] where two sessions were scheduled per week. Different methods like discussion, roleplaying, question and answer sessions and visual media like films and images were used to provide this educational content.

### Risk-of-bias assessment

The risk of bias assessment for the randomised controlled trials [45,46,48] using RoB 2.0 demonstrated some concerns/unclear risk of bias for two studies and high risk of bias in one study (**Table 4**). In the study by Saffari et al. [45], all the domains had low risk of bias except for the randomisation process, which received a “some concerns” rating because the method of random allocation sequence was not reported; while this did not give rise to any baseline differences, it did affect the overall rating of the study. The study by Askari et al. [46] had two domains rated as having “some concerns” – the randomisation process, where information on random allocation sequence was unavailable, and the effect of adhering to intervention raised some concerns, as there was no information about the co-interventions. This led to the overall quality being determined as having “some concerns”. Study by Karimi et al. [48] had an overall high risk of bias due to alternation during random sequence generation (rated as “high” risk of bias) and “some concerns” regarding the selection of the reported result.

**Table 4:** Risk of bias for randomised studies using Cochrane’s risk of bias tool version 2018 (Rob 2.0)

Study	Randomisation process	Effect of assignment to intervention	Effect of adhering to intervention	Missing outcome data	Measurement of outcome data	Selection of reported result	Overall risk of bias
Saffari et al [45]	Method not disclosed†	Participants and personnel unaware and appropriate analysis used*	Participants and personnel were unaware, no information on co-interventions but outcome unaffected; participants adhered to assigned intervention and appropriate analysis used*	Outcome data for all participants not available but results not biased by missing outcome data*	Appropriate method of measuring outcome and outcome wouldn’t have differed; assessors aware but outcome not influenced by knowledge of intervention received*	Trial analysed as per pre-specified plan; numerical result assessed unlikely to be have been selected from multiple outcomes and multiple analysis*	Some concerns
Askari et al. [46]	Method not disclosed†	Participants unaware, personnel aware but no deviation from intended intervention and appropriate analysis used*	Participants unaware, personnel aware, no information on co-interventions but outcome unaffected; participants adhered to assigned intervention and appropriate analysis used*	Outcome data for all participants available*	Appropriate method of measuring outcome and outcome wouldn’t have differed; assessors unaware of intervention received*	Trial analysed as per pre-specified plan; numerical result assessed unlikely to be have been selected from multiple outcomes and multiple analysis*	Some concerns
Karimy et al. [48]	Assignment between groups as per alternation‡	Participants unaware, personnel – no information but no deviation from intended intervention and appropriate analysis used*	Participants unaware, personnel – no information; co-intervention balanced and outcome unaffected and participants adhered to assigned intervention*	Outcome data for all participants not available but results not biased by missing outcome data*	Appropriate method of measuring outcome and outcome wouldn’t have differed; assessors unaware of intervention received*	No information on pre-specified plan but numerical result assessed unlikely to have been selected from multiple outcomes and multiple analysis*	High risk

*Low risk of bias.

†Some concerns.

‡High risk of bias.

Regarding the non-randomised study [47], outcome accessors were probably aware of the intervention received by the study participants and could have influenced the outcomes, although there were no systematic errors in measurement of the outcome related to intervention received, leading to an assessment of “moderate” for the overall risk of bias (**Table 5**). Overall, one study had a high risk of bias and other three had some concerns/moderate risk of bias.

**Table 5:** Risk of bias for non-randomised studies using Cochrane’s Risk of Bias in Non-randomised Studies – of Interventions (ROBINS-I) tool

Study	Confounding	Selection of participants	Classification of interventions	Deviation from intended interventions	Missing outcome data	Measurement of outcomes	Reporting of results	Overall risk of bias	
Jeihooni et al. [47]	No potential of confounding effect*	Selection of participants not based on participants characteristics observed after start of intervention; follow-up and start of intervention coincide for most participants*	Intervention groups were clearly defined and recorded at the start of the intervention*	No deviations from intended intervention beyond expected in usual practice*	Outcome data available for all participants†	Outcome measure could have been influenced by knowledge of intervention received and assessors were aware of intervention but methods of outcome assessment comparable across groups and there were no systematic errors in measurement†	Reported effect estimate unlikely to be selected on basis of multiple outcomes or multiple analyses and subgroups*	Moderate risk of bias

*Low risk of bias

†Moderate risk of bias.

## DISCUSSION

We conducted this SR as part of the updated MRC guidance on complex intervention development and evaluation [59], which seeks to develop an intervention by identifying a theory and generating an evidence base. The TPB framework has increasingly been used for developing and evaluating behaviour change interventions [19] and for designing interventions for improving treatment adherence, avoidance of risk behaviours, and advancing follow-up to improve health outcomes [60,61]. Through this SR, we sought to gather evidence on feasibility of TPB interventions in low-resource, low-literacy settings, and to inform its effectiveness using the available resources (methods, modes of delivery, and health personnel) in such settings. Studies that met the inclusion criteria were reviewed for quality and included in the narrative synthesis, so that recommendations for those in the process of designing and evaluating studies could be made.

### Study setting, design and delivery

Our objective was to describe the study settings and the way the intervention was delivered. All studies were from a single country (defined as an LMIC by the World bank [41]), and were conducted among the urban population residing in cities. One study was hospital-based [45] and the others were community-based studies [46-48]. These were interventional or quasi-experimental studies with intervention and control groups for which the intervention was designed on TPB constructs to change attitudes towards behaviour, subjective norms, perceived behavioural control, intention and behaviour; one study [45] additionally looked at improving clinical outcomes and quality of life. All studies had a similar mode of intervention delivery, with group education being the preferred option; education was given face-to-face in all studies except for one which opted for the hybrid option [46]. The studies were not precise in describing the health providers who delivered the intervention, with terms like trainer [45], researcher [46], educator [47], and instructor [48] being used for those providing the education; there was no mention of whether they were part of the health system or their roles within the health care network. One study [47] specified a cardiologist and nutritionist as those providing one of the sessions of health education, but this was the exception. Different media and methods were used for providing the educational interventions, the common being group discussions, educational videos or films, question and answers sessions, and brainstorming; other methods used were booklets, CDs, educational movies, and role-playing.

These studies provide insigths into the study setting (i.e. location, type of group chosen) and into details about the intervention delivery. Although all the studies were on urban populations, a significant proportion had low educational levels. They did not provide any information about the rurality of the setting, and the study designs did not describe the use of cluster randomisation. The methods and media of the intervention were usually quite clearly described, but their providers and roles were not.

### Feasibility and fidelity of the interventions

We conducted this SR to assess the feasibility, i.e. the impact an intervention has on its end user and the resources required to successfully implement the intervention [62], and the fidelity, i.e. the degree to which an intervention has been delivered as intended [63]. Feasibility looks at whether an intervention is appropriate, can be implemented in a particular setting, and whether it is relevant and sustainable. To evaluate feasibility, we looked at recruitment rates, the integrity of intervention delivery, the completion rates, and the timescale of completion to evaluate. Two studies have high recruitment rates [45,46], while the other two [47,48] had no mention of recruitment rates, but had achieved their target sample sizes. We assessed the integrity of the intervention delivery by whether there was a structured method to intervention, its implementation, and whether it reached the intended participants. All studies had a structured intervention and followed the time schedule for its delivery to the intervention arm group; furthermore, the media and methods were clearly described and all were completed within the stipulated time frame. The controls had either standard education as per routine practice or had no education during the intervention period; some were given educational materials at the end of the intervention period to maintain research ethics [47]. Two studies had 100% completion rates [46,47] and other two [45,48] had completion rates close to 90%, demonstration the acceptability among the participants and the fidelity of the intervention. Overall, the interventions demonstrated feasibility and the fidelity and provided researchers with insights to develop and deliver such interventions in these settings.

### Impact of the interventions

All the four studies demonstrated the effectiveness of the interventions in terms of improvement in TPB constructs, measured quantitatively through mean changes and their significance (*P* < 0.05). Three studies [45,47,48] showed significant improvement (*P* < 0.05) in all five TPB constructs following the intervention, while one [46] showed significant improvement in attitude, subjective norms, and behaviour. There was significant improvement (*P* < 0.05) in health behaviour (nutrition, exercise-jogging, lifestyle), clinical outcomes, and quality of life following the intervention, and one study [47] showed that TPB constructs were responsible for 39.6% change in intention towards a lifestyle. All the studies demonstrated that desired outcomes could be achieved by implementing TPB-based interventions, in diverse chronic conditions ranging from cardiovascular disease to diabetes and osteoarthritis. The total study duration varied from five months to a year, but the intervention period for each study lasted for one month, with the evaluation being done three months after the completion of the intervention. The impact of the intervention was effective over a short duration, but there was no information about its effect on long term or the effect of an intervention carried out over a longer period.

### Methodological quality of studies

Three studies were randomised controlled trials and one was of a quasi-experimental design, suggesting that robust study designs were used for testing the interventions and therefore the outcomes were reliable and may be replicated in similar settings.

Assessing the quality of studies, two of three randomised controlled trials [45,46] had some concerns, as allocation sequence during the randomisation process was not mentioned, while the third [48] had a high risk of bias as the participants were assigned by alternation, making assignment predictable and subject to bias. Only one study [45] used the Consolidated Standard for Reporting Trials (CONSORT) 2010 updated guidelines [64]; this use of reporting guidelines improves methodological rigour, reporting quality, and prevents bias, while allowing researchers to study and replicate such trials in other settings.

With this SR, we examined studies from the LMIC settings to provide valuable inputs about the feasibility, impact, and methods of intervention delivery. It also underlined the need for conducting more such studies as currently the limited number of studies do not provide complete information for development and implementation of such interventions in a diverse array of chronic conditions.

### Strengths and limitations

This SR included studies with robust study designs, with quantitatively described outcomes through estimates and strength of significance (*P*-values), and well-described interventions. It is the first to provide information on the feasibility, effectiveness, and methods of implementing TPB interventions in chronic disease patients in low-health literacy settings. We used PRISMA-P guidelines to develop the protocol [65] and register it at PROSPERO, and used PRISMA 2020 guidance on reporting [58] with two reviewers screening titles, abstracts, and full papers. However, we included only four studies and were unable to provide the expected diversity of information about different kinds of population characteristics or study settings, as all the studies were from a single country and from an urban background. Moreover, the studies were not high-quality: three were of moderate quality with some concerns or with a moderate risk of bias, providing some degree of reliability of the information.

## CONCLUSIONS

While TPB is effective in changing health behaviour, there was little evidence of the feasibility and applicability of such interventions in different settings, particularly in LMICs. Through this SR, we provided evidence of effectiveness of TPB interventions as well as their feasibility in such settings. This provides some insights on developing and implementing TPB based interventions with regards to setting, time frame, media and methods. Such interventions are limited in LMICs, where health behaviour change can be an effective and economic tool for fighting chronic diseases, therefore more such studies are required to gather definitive evidence and prove their feasibility and utility in LMICs.

## Additional material


Online Supplementary Document
