# Supplementary material for: A systematic review of the theory of planned behaviour interventions for chronic diseases in low health-literacy settings
Source: J Glob Health. 2023 Sep 8;13:04079. doi: 10.7189/jogh.13.04079 (PMC10506128; doi:10.7189/jogh.13.04079)
Supplement: Online Supplementary Document [file jogh-13-04079-s001.pdf]

Additional File 1

Additional File 1 Search Strategy MEDLINE

1. exp Psychological Theory/ or theory of planned behaviour.mp.
2. TPB.mp.
3. 1 or 2
4. theory of reasoned action.mp.
5. TRA.mp.
6. 4 or 5
7. behavioural control.mp.
8. control.mp. or exp BEHAVIOR CONTROL/
9. 6 and 7
10. 6 and 8
11. 9 or 10
12. 3 or 11
13. belief*.mp.
14. exp ATTITUDE TO HEALTH/ or exp ATTITUDE/ or attitude.mp.
15. attitude to behaviour.mp.
16. 14 or 15
17. (subjective norm or social norm).af.
18. 7 or 8
19. 13 and 16 and 17 and 18
20. (Ajzen * not Fishbein *).au.
21. self-efficacy.mp. or exp Self Efficacy/
22. exp INTENTION/ or intention.mp.
23. 13 and 18 and 21 and 22
24. 12 or 19 or 20 or 23
25. intervention studies.mp.
26. clinical trials.mp. or exp Clinical Trial/
27. evaluation studies.mp. or exp Evaluation Studies/
28. multicentre study.mp.
29. programme evaluation.mp. or exp Programme Evaluation/
30. exp Randomised Controlled Trials as Topic/ or randomised controlled trials.mp.
31. non-randomised controlled trials.mp.

32. follow-up studies.mp. or exp Follow-Up Studies/
33. longitudinal studies.mp. or exp Longitudinal Studies/
34. feasibility studies.mp. or exp Feasibility Studies/
35. 25 or 26 or 27 or 28 or 29 or 30 or 31 or 32 or 33 or 34
36. 24 and 35
37. behaviour change.mp.
38. health behaviour change.mp.
39. exp Health Behavior/ or health behaviour.mp.
40. 37 or 38 or 39
41. chronic disease.mp. or exp Chronic Disease/
42. non-communicable diseases.mp. or exp Noncommunicable Diseases/
43. exp Diabetes Mellitus/ or chronic conditions.mp.
44. chronic illness.mp.
45. exp Asthma/ or exp Pulmonary Disease, Chronic Obstructive/ or exp Lung Diseases/ or chronic respiratory diseases.mp. or exp Lung Diseases, Obstructive/ or lung cancer.mp. or exp Lung Neoplasms/
46. 41 or 42 or 43 or 44 or 45
47. adherence.mp. or exp GUIDELINE ADHERENCE/ or exp "TREATMENT ADHERENCE AND COMPLIANCE"/ or exp MEDICATION ADHERENCE/
48. compliance.mp. or exp COMPLIANCE/ or exp PATIENT COMPLIANCE/
49. concordance.mp.
50. 47 or 48 or 49
51. 40 or 50
52. 36 and 51
53. 46 and 52
54. 36 and 46
55. (Africa or Asia or Caribbean or West Indies or South America or Latin America or Central America or Afghanistan or Albania or Algeria or Angola or Antigua or Barbuda or Argentina or Armenia or Armenian or Aruba or Azerbaijan or Bahrain or Bangladesh or Barbados or Benin or Byelarus or Byelorussian or Belarus or Belorussian or Belorussia or Belize or Bhutan or Bolivia or Bosnia or Herzegovina or Hercegovina or Botswana or Brasil or Brazil or Bulgaria or Burkina Faso or Burkina Fasso or Upper Volta or Burundi or Urundi or Cambodia or Khmer Republic or Kampuchea or Cameroon or Cameroons or Cameron or Camerons or Cape Verde or Central African Republic or Chad or Chile or China or Colombia or Comoros or Comoro Islands or Comores or Mayotte or Congo or Zaire or Costa Rica or Cote d'Ivoire or Ivory Coast

or Croatia or Cuba or Cyprus or Czechoslovakia or Czech Republic or Slovakia or Slovak Republic or Djibouti or French Somaliland or Dominica or Dominican Republic or East Timor or East Timur or Timor Leste or Ecuador or Egypt or United Arab Republic or El Salvador or Eritrea or Estonia or Ethiopia or Fiji or Gabon or Gabonese Republic or Gambia or Gaza or Georgia or Georgian or Ghana or Gold Coast or Greece or Grenada or Guatemala or Guinea or Guam or Guiana or Guyana or Haiti or Honduras or Hungary or India or Maldives or Indonesia or Iran or Iraq or Isle of Man or Jamaica or Jordan or Kazakhstan or Kazakh or Kenya or Kiribati or Korea or Kosovo or Kyrgyzstan or Kirghizia or Kyrgyz Republic or Kirghiz or Kirgizstan or Lao PDR or Laos or Latvia or Lebanon or Lesotho or Basutoland or Liberia or Libya or Lithuania or Macedonia or Madagascar or Malagasy Republic or Malaysia or Malaya or Malay or Sabah or Sarawak or Malawi or Nyasaland or Mali or Malta or Marshall Islands or Mauritania or Mauritius or Agalega Islands or Mexico or Micronesia or Middle East or Moldova or Moldovia or Moldovian or Mongolia or Montenegro or Morocco or Ifni or Mozambique or Myanmar or Myanma or Burma or Namibia or Nepal or Netherlands Antilles or New Caledonia or Nicaragua or Niger or Nigeria or Northern Mariana Islands or Oman or Muscat or Pakistan or Palau or Palestine or Panama or Paraguay or Peru or Philippines or Philipines or Phillipines or Phillippines or Poland or Portugal or Puerto Rico or Romania or Rumania or Roumania or Russia or Russian or Rwanda or Ruanda or Saint Kitts or St Kitts or Nevis or Saint Lucia or St Lucia or Saint Vincent or St Vincent or Grenadines or Samoa or Samoan Islands or Navigator Island or Navigator Islands or Sao Tome or Saudi Arabia or Senegal or Serbia or Montenegro or Seychelles or Sierra Leone or Slovenia or Sri Lanka or Ceylon or Solomon Islands or Somalia or Sudan or Suriname or Surinam or Swaziland or Syria or Tajikistan or Tadzhikistan or Tadjikistan or Tadzhik or Tanzania or Thailand or Togo or Togolese Republic or Tonga or Trinidad or Tobago or Tunisia or Turkey or Turkmenistan or Turkmen or Uganda or Ukraine or Uruguay or USSR or Soviet Union or Union of Soviet Socialist Republics or Uzbekistan or Uzbek or Vanuatu or New Hebrides or Venezuela or Vietnam or Viet Nam or West Bank or Yemen or Yugoslavia or Zambia or Zimbabwe or Rhodesia).mp.
56. DEVELOPING COUNTRIES/
57. ((developing or less* developed or under developed or underdeveloped or middle income or low* income or underserved or under served or deprived or poor*) adj3 (countr* or nation* or population* or world)).mp.
58. ((developing or less* developed or under developed or underdeveloped or middle income or low* income) adj1 (economy or economies)).mp.
59. (low* adj1 (GDP or GNP or gross domestic or gross national)).mp.
60. (low adj3 middle adj3 countr*).mp.
61. (LMIC or LMICs or third world or LAMI country or LAMI countries).mp.
62. (transitional country or transitional countries).mp.

63. or/55-62
64. 53 and 63
65. 35 or 51
66. 24 and 46 and 63 and 65

Additional File 2

Data extraction form and Robbins-II risk of bias tool

Data extraction form

Identification

Review title or ID	
Study ID *(surname of first author and year first full report of study was published eg Smith 2001)*	
Report ID	
Report ID of other reports of this study including errata or retractions	
Notes	

General Information

Date form completed *(dd/mm/yyyy)*	
Name/ID of person extracting data	
Reference citation	
Study author contact details	
Publication type *(eg full report, abstract, letter)*	
Notes:	

Study Eligibility

Study Characteristics	Eligibility criteria *(Insert inclusion criteria for each characteristic as defined in the Protocol)*	Eligibility criteria met? Yes No Unclear	Location in text or source *(pg & ¶/fig/table/other)*
Type of study	Randomised Controlled Trial	☐ ☐ ☐	
	Quasi-randomised Controlled Trial	☐ ☐ ☐	
	Cluster Randomised Trial	☐ ☐ ☐	
	Controlled Before and After Study<br>Contemporaneous data collection<br>Comparable control sites<br>At least 2 x intervention and 2 x control clusters	☐ ☐ ☐	

	Interrupted Time Series<br>At least 3 time points before and 3 after the intervention<br>Clearly defined intervention point	☐ ☐ ☐	
	Other design (specify):	☐ ☐ ☐	
Types of intervention	TPB based intervention	☐ ☐ ☐	
	At least 2 of the 3 constructs of TPB used by the intervention	☐ ☐ ☐	
	Intervention using multiple psychological theories with clearly measurable TPB constructs	☐ ☐ ☐	
Types of comparison / control	Health education not based on any psychological theory	☐ ☐ ☐	
	Health education based on psychological theory other than TPB	☐ ☐ ☐	
	Treatment as usual without any structured health education	☐ ☐ ☐	
Participants	Adults above 18 years of age, any gender and not Caucasian<br>Should have any chronic disease	☐ ☐ ☐	
Study setting	The geographical location of the study should be a LMIC	☐ ☐ ☐	
Types of outcome measures	Knowledge	☐ ☐ ☐	
	Attitude, subjective norms, perceived behavioural control	☐ ☐ ☐	
	Health behaviour *(eg exercise, medication use, smoking cessation, inhaler use)*	☐ ☐ ☐	
INCLUDE ☐		EXCLUDE ☐	
Reason for exclusion			
Notes:			

DO NOT PROCEED IF STUDY EXCLUDED FROM REVIEW

Characteristics of included studies

Methods

	**Descriptions as stated in report/paper**		**Location in text or source** *(pg & ¶/fig/table/other)*
**Aim of study** *(eg efficacy, equivalence, pragmatic)*			
**Design** *(eg parallel, crossover, non-RCT)*			
**Unit of allocation** *(by individuals, cluster/ groups or body parts)*			
**Start date**			
**End date**			
**Duration of participation** *(from recruitment to last follow-up)*			
**Ethical approval needed/ obtained for study**	☐ Yes ☐ No ☐ Unclear		
**Notes:**			

Participants

	Description *Include comparative information for each intervention or comparison group if available*	Location in text or source *(pg & ¶/fig/table/other)*

Population description *(from which study participants are drawn)*		
Setting *(including location and social context)*		
Inclusion criteria		
Exclusion criteria		
Method of recruitment of participants *(eg phone, mail, clinic patients)*		
Informed consent obtained	☐ Yes ☐ No ☐ Unclear	
Total no. randomised *(or total pop. at start of study for NRCTs)*		
Clusters *(if applicable, no., type, no. people per cluster)*		
Baseline imbalances		
Withdrawals and exclusions *(if not provided below by outcome)*		
Age		
Sex		
Race/Ethnicity		
Severity of illness		
Co-morbidities		
Other relevant socio-demographics		
Subgroups measure		
Subgroups reported		
Notes:		

Intervention groups

Copy and paste table for each intervention and comparison group

Intervention Group 1

	Description as stated in report/paper	Location in text or source *(pg & ¶/fig/table/other)*
Group name		
No. randomised to group *(specify whether no. people or clusters)*		
Theoretical basis *(include key references)*		
Description *(include sufficient detail for replication, eg content, dose, components)*		
Duration of treatment period		
Timing *(eg frequency, duration of each episode)*		
Delivery *(eg mechanism, medium, intensity, fidelity)*		
Providers *(eg no., profession, training, ethnicity etc. if relevant)*		
Co-interventions		
Economic information *(ie intervention cost, changes in other costs as result of intervention)*		
Resource requirements *(eg staff numbers, cold chain, equipment)*		
Integrity of delivery		
Compliance		
Notes:		

Outcomes

Copy and paste table for each outcome.

Outcome 1

	Description as stated in report/paper	Location in text or source *(pg & ¶/fig/table/other)*
Outcome name		
Time points measured *(specify whether from start or end of intervention)*		
Time points reported		
Outcome definition *(with diagnostic criteria if relevant)*		
Person measuring/ reporting		
Unit of measurement *(if relevant)*		
Scales: upper and lower limits *(indicate whether high or low score is good)*		
Is outcome/tool validated?	☐ Yes ☐ No ☐ Unclear	
Imputation of missing data *(eg assumptions made for ITT analysis)*		
Assumed risk estimate *(eg baseline or population risk noted in Background)*		

Power *(eg power & sample size calculation, level of power achieved)*		
Notes:		

Other

**Study funding sources** *(including role of funders)*		
**Possible conflicts of interest** *(for study authors)*		
**Notes:**		

ROBBINS-II ***Risk of Bias assessment tool***

Domain	Risk of bias<br>Low High Unclear	Support for judgement<br>*(include direct quotes where available with explanatory comments)*	Location in text or source *(pg & ¶/fig/table/other)*
Random sequence generation *(selection bias)*	☐ ☐ ☐		
Allocation concealment *(selection bias)*	☐ ☐ ☐		
Blinding of participants and personnel *(performance bias)*	☐ ☐ ☐	Outcome group: All/	
(if separate judgement by outcome(s) required)	☐ ☐ ☐	Outcome group:	
Blinding of outcome assessment *(detection bias)*	☐ ☐ ☐	Outcome group: All	
(if separate judgement by outcome(s) required)	☐ ☐ ☐	Outcome group:	
Incomplete outcome data *(attrition bias)*	☐ ☐ ☐	Outcome group: All/	
(if separate judgement by outcome(s) required)	☐ ☐ ☐	Outcome group:	
Selective outcome reporting? *(reporting bias)*	☐ ☐ ☐		

Other bias	☐ ☐ ☐		
Notes:			

Data and analysis

For RCT/CCT

Dichotomous outcome

	Description as stated in report/paper				Location in text or source *(pg & ¶/fig/table/other)*
Comparison					
Outcome					
Subgroup					
Time point *(specify from start or end of intervention)*					
Results	Intervention		Comparison		
	No. with event	Total in group	No. with event	Total in group	
Any other results reported *(eg odds ratio, risk difference, CI or P value)*					
No. missing participants					
Reasons missing					
No. participants moved from other group					
Reasons moved					
Unit of analysis *(by individuals, cluster/groups or body parts)*					

Statistical methods used and appropriateness of these *(eg adjustment for correlation)*			
Reanalysis required? *(specify, eg correlation adjustment)*	☐ Yes ☐ No ☐ Unclear		
Reanalysis possible?	☐ Yes ☐ No ☐ Unclear		
Reanalysed results			
Notes:			

For RCT/CCT

Continuous outcome

	Description as stated in report/paper						Location in text or source *(pg & ¶/fig/table/other)*
Comparison							
Outcome							
Subgroup							
Time point *(specify from start or end of intervention)*							
Post-intervention or change from baseline?							
Results	Intervention			Comparison			
	Mean	SD *(or other variance, specify)*	No. participants	Mean	SD *(or other variance, specify)*	No. participants	
Any other results reported *(eg mean difference, CI, P value)*							

No. missing participants			
Reasons missing			
No. participants moved from other group			
Reasons moved			
Unit of analysis *(individuals, cluster/ groups or body parts)*			
Statistical methods used and appropriateness of these *(eg adjustment for correlation)*			
Reanalysis required? *(specify)*	☐ Yes ☐ No ☐ Unclear		
Reanalysis possible?	☐ Yes ☐ No ☐ Unclear		
Reanalysed results			
Notes:			

For RCT/CCT

Other outcome

	Description as stated in report/paper		Location in text or source *(pg & ¶/fig/table/other)*
Comparison			
Outcome			
Subgroup			
Time point *(specify from start or end of intervention)*			
No. participant	Intervention	Control	

Results	Intervention result	SE (or other variance)	Control result	SE (or other variance)	
	Overall results		SE (or other variance)		
Any other results reported					
No. missing participants					
Reasons missing					
No. participants moved from other group					
Reasons moved					
Unit of analysis *(by individuals, cluster/groups or body parts)*					
Statistical methods used and appropriateness of these					
Reanalysis required? *(specify)*	☐ Yes ☐ No ☐ Unclear				
Reanalysis possible?	☐ Yes ☐ No ☐ Unclear				
Reanalysed results					
Notes:					

For Controlled Before-and-After study (CBA)

	Description as stated in report/paper	Location in text or source *(pg & ¶/fig/table/other)*
Comparison		

<table>
<tr><td>Outcome</td><td colspan="4"></td><td></td></tr>
<tr><td>Subgroup</td><td colspan="4"></td><td></td></tr>
<tr><td>Time point (specify from start or end of intervention)</td><td colspan="4"></td><td></td></tr>
<tr><td>Post-intervention or change from baseline?</td><td colspan="4"></td><td></td></tr>
<tr><td rowspan="2">No. participants</td><td colspan="2">Intervention</td><td colspan="2">Control</td><td rowspan="2"></td></tr>
<tr><td colspan="2"></td><td colspan="2"></td></tr>
<tr><td rowspan="4">Results</td><td>Intervention result</td><td>SE (or other variance, specify)</td><td>Control result</td><td>SE (or other variance, specify)</td><td rowspan="4"></td></tr>
<tr><td></td><td></td><td></td><td></td></tr>
<tr><td colspan="2">Overall results</td><td colspan="2">SE (or other variance, specify)</td></tr>
<tr><td colspan="2"></td><td colspan="2"></td></tr>
<tr><td>Any other results reported</td><td colspan="4"></td><td></td></tr>
<tr><td>No. missing participants</td><td colspan="2"></td><td colspan="2"></td><td></td></tr>
<tr><td>Reasons missing</td><td colspan="2"></td><td colspan="2"></td><td></td></tr>
<tr><td>No. participants moved from other group</td><td colspan="2"></td><td colspan="2"></td><td></td></tr>
<tr><td>Reasons moved</td><td colspan="2"></td><td colspan="2"></td><td></td></tr>
<tr><td>Unit of analysis (individuals, cluster/ groups or body parts)</td><td colspan="4"></td><td></td></tr>
<tr><td>Statistical methods used and appropriateness of these</td><td colspan="4"></td><td></td></tr>
<tr><td>Reanalysis required? (specify)</td><td colspan="2">☐ ☐ ☐
Yes No Unclear</td><td colspan="2"></td><td></td></tr>
</table>

Reanalysis possible?	☐ ☐ ☐<br>Yes No<br>Unclear		
Reanalysed results			
Notes:			

Other information

	**Description as stated in report/paper**	**Location in text or source** *(pg & ¶/fig/table/other)*
**Key conclusions of study authors**		
**References to other relevant studies**		
**Correspondence required for further study information** *(from whom, what and when)*		
**Notes:**		
